# Systematic study of the synergistic and kinetics effects on the removal of contaminants of emerging concern from water by ultrasound in the presence of diverse oxidants

**DOI:** 10.1007/s11356-023-29189-y

**Published:** 2023-08-26

**Authors:** Sandra E. Estrada-Flórez, Efraím A. Serna-Galvis, Judy Lee, Ricardo A. Torres-Palma

**Affiliations:** 1 Grupo de Investigación en Remediación Ambiental y Biocatálisis (GIRAB), Instituto de Química, Facultad de Ciencias Exactas y Naturales, Universidad de Antioquia UdeA, Calle 70 No. 52-21, Medellín, Colombia; 2 Grupo de Catalizadores y Adsorbentes (CATALAD), Instituto de Química, Facultad de Ciencias Exactas y Naturales, Universidad de Antioquia UdeA, Calle 70 No. 52-21, Medellín, Colombia; 3 School of Chemistry and Chemical Engineering, University of Surrey, Guildford, GU2 7XH United Kingdom

**Keywords:** Ultrasound, Acetaminophen, Hydrogen peroxide, Peroxydisulfate, Peroxymonosulfate, Water treatment

## Abstract

The enhancement of the ultrasound system by adding diverse oxidants to remove a model contaminant (acetaminophen, ACE) in water was investigated. Different parameters were evaluated to study their effect on both the degradation kinetics and the synergy of the combination. The variables studied were the ultrasonic frequency (575, 858, and 1135 kHz), type of oxidant (hydrogen peroxide, sodium peroxydisulfate (or persulfate, PDS), and potassium peroxymonosulfate (PMS)), ACE concentration (4, 8, and 40 µM), and oxidant concentration (0.01, 0.1, 1, and 5 mM). Particular interest was placed on synergistic effects, implying that one process (or both) is activated by the other to lead to greater efficiency. Interestingly, the parameters that led to the higher synergistic effects did not always lead to the most favorable degradation kinetics. An increase in ACE removal of 20% was obtained using the highest frequency studied (1135 kHz), PMS 0.1 mM, and the highest concentration of ACE (40 µM). The intensification of degradation was mainly due to the ability of ultrasound to activate oxidants and produce extra hydroxyl radicals (HO^•^) or sulfate radicals (SO_4_^•–^). Under these conditions, treatment of ACE spiked into seawater, hospital wastewater, and urine was performed. The hospital wastewater matrix inhibited ACE degradation slightly, while the urine components inhibited the pollutant degradation completely. The inhibition was mainly attributed to the competing organic matter in the effluents for the sono-generated radical species. On the contrary, the removal of ACE in seawater was significantly intensified due to “salting out” effects and the production of the strong oxidant HOCl from the reaction of chloride ions with PMS.

**Supplementary Information:**

The online version contains supplementary material available at 10.1007/s11356-023-29189-y.

## Introduction

Contaminants of emerging concern (CECs) include pharmaceuticals (PhPs), cosmetics, synthetic and natural hormones, endocrine disruptors, disinfection by-products, biocides, etc. (Barbosa et al. 2016; Dey et al. 2019; Patel et al. 2019). CECs are released from chemical, pharmaceutical, agricultural industries, hospitals, and domestic wastewater (Bilal et al. 2019; Ohoro et al. 2019). Removing CECs by conventional methods in wastewater treatment plants (WWTP) is challenging. Therefore, CECs reach natural waters (e.g., surface water or seawater), causing problems not only to human health but also triggering environmental issues such as toxicity in the aquatic environment, and the proliferation of bacterial resistance, among others (Jelic et al. 2011; Gracia-Lor et al. 2012; Geissen et al. 2015; Manaia et al. 2016; Kurwadkar 2019). Because of this, many researchers have focused their studies on advanced oxidation processes (AOPs) to effectively degrade CECs in water matrices (Bartolomeu et al. 2018; Miklos et al. 2018; Rizzo et al. 2019).

Ultrasound technique (US), especially high-frequency US, is an AOP widely studied for CECs removal (Rayaroth et al. 2016; Serna-Galvis et al. 2019b, a; Meng et al. 2019; Estrada-Flórez et al. 2020). The US process is based on the acoustic cavitation phenomenon, i.e., the ultrasound waves induce the formation and growth of bubbles or cavities from dissolved gas. These cavities reach a critical size, and then they collapse, generating strong conditions (~ 5000 K, ~ 1000 atm), which promote the dissociation of dissolved oxygen and water molecules producing hydroxyl radicals (HO^•^). In recent years, several strategies have been reported to intensify the degradation of pollutants by US. The combination of US with light (sono-photolysis or photo-sonolysis) (Patidar and Srivastava 2021), iron or iron/oxidants (sono-Fenton process), or iron and light (sono-photo-Fenton process) (Barzegar et al. 2018; Prada-Vásquez et al. 2021; Cui et al. 2021a; Patil and Raut-Jadhav 2022), its combination with photocatalysis (sono-photo-catalysis) (Stucchi et al. 2019), or even sono-Fenton mediated by TiO_2_-P25 photocatalysis (Xu et al. 2020b; Qi et al. 2020) are among the most outstanding alternatives. Also, the combination of US with carbonaceous materials has been explored (Diao et al. 2020; Grilla et al. 2020).

Recently, the addition of oxidants such as hydrogen peroxide, potassium peroxymonosulfate (PMS), or sodium/potassium persulfate (PDS) to the US process has gained the attention of the scientific community (Lim et al. 2014; Xu et al. 2020a; Gujar et al. 2021; Lee et al. 2021; Moradnia et al. 2022). The addition of H_2_O_2_, PMS, or PDS to ultrasound (US/Oxidant system) can enhance the capacity of US to generate SO_4_^•^ and extra HO^•^ from the cleavages of these oxidants. Indeed, some works have shown that the US/Oxidant systems are environmentally friendly and highly effective for the treatment of organic pollutants (Raut-Jadhav et al. 2016; Lee et al. 2021). In the combination of such processes, a synergistic effect is generally expected, which implies that one of the processes is positively affected by the other, or that each process is activated by the other one to improve the degradation efficiency of the target pollutant. However, studies usually focus on kinetics or synergy independently and few investigations have contrasted the kinetic and synergistic aspects of the US/Oxidant combination. The ideal scenario in the combinations is that both kinetic and synergy values are high and lead to efficient processes (Lim et al. 2014; Xu et al. 2020a; Cui et al. 2021b; Ioannidi et al. 2022; Patil and Raut-Jadhav 2022). The question that arises is: are the highly synergistic systems the most kinetically favored? This has not been answered yet, and our study pretends to address it.

In this work, we started evaluating fundamental aspects of the high-frequency ultrasound in combination with H_2_O_2_, PMS, and PDS, with a focus on both synergistic and degradation kinetics, using acetaminophen (ACE), also called paracetamol, as the model contaminant (see Text S1 and Table S1 in Supplementary material). The following parameters were tested: (i) ultrasonic frequency, (ii) type of oxidant, (iii) pollutant concentration, and (iv) oxidant concentration. Furthermore, parameters that led to the best synergistic effects were selected for a subsequent evaluation in the degradation of ACE spiked into various world-real matrices: urine, hospital wastewater (HWW), and seawater (SW). Urine was selected because it constitutes one of the main routes of excretion of pharmaceutical compounds. In the same way, HWW is also one of the main receiving sources of these wastes. Moreover, HWW in some coastal areas is discharged directly into the sea. Thus, SW was also selected as a probe matrix.

## Materials and methods

### Reagents

Acetaminophen (ACE) was provided by Laproff (Medellín, Colombia). Sodium peroxydisulfate (PDS, Na_2_S_2_O_8_) was purchased from Fisher Scientific (England, UK), and Oxone (KHSO_5_·0.5KHSO_4_·0.5K_2_SO_4_), which is the source of potassium peroxymonosulfate (PMS, KHSO_5_), was supplied by Sigma Aldrich (St Louis, USA). Hydrogen peroxide (H_2_O_2_) 30% (w/v), ammonium heptamolybdate (AHM), sodium bicarbonate (NaHCO_3_), and solvents for HPLC (analytical grade acetonitrile (MeCN) and methanol (MeOH)) were purchased from Merck (Darmstadt, Germany). Potassium iodide (KI) was supplied by Panreac (Barcelona, Spain). Formic acid (HCOOH) was acquired from Carlo-Erba (Val-de-Reuil, France). All chemicals were used as received without further purification. Distilled water (DW) was used for initial pharmaceutical solutions preparation. Milli-Q water was employed for HPLC analyses. All mobile phases were filtered through 0.45 μm nylon or using mixed cellulose-ester filters (Advantec).

For the experiments in actual matrices, a sample of seawater (SW) collected from the pacific sea (Tumaco, Colombia) in July 2021 was used. A hospital wastewater sample (HWW) was taken in April 2019 from the effluent of a health center from Tumaco (Colombia) during a typical day of hospital operation. Furthermore, a real fresh urine sample collected in December 2021 from a healthy and no medicated person was used. The complete characterization of the HWW can be found in the literature (Serna-Galvis et al. 2019c, 2022). All samples were kept refrigerated at 4 °C until use and filtered (0.45 μm nylon Advantec filters) before experiments.

### Methods

A Meinhardt ultrasound reactor with a cooling jacket and a maximum capacity of 500 mL was utilized for the sonochemical experiments. The reactor was connected to a Huber-Minichiller thermostatic bath, which was adjusted to keep a temperature of 20 ± 3 °C inside the reactor. The ultrasonic device was adjusted to the desired frequency through different transducers. The actual ultrasonic power densities at the different frequencies were measured by the calorimetric method (Text S2, Fig. S1, and Table S2) (Kimura et al. 1996). A scheme of the reactor is shown in Fig. S2.

Degradation experiments were carried out individually: (a) direct oxidation with selected oxidants (PMS, H_2_O_2_, or PDS) under mild stirring, and (b) sonolysis at the selected frequencies. Subsequently, the degradation on the combined system was evaluated: (c) US/Oxidant process at the different frequencies and selected oxidant concentrations. The synergy index (*S*) for the US/Oxidant system was determined using Eq. 1 (Torres-Palma et al. 2010).

1 $$S=\frac{{k}_{US/Oxidant}}{{k}_{US}+{k}_{Oxidant}}$$

where *k*_*US/Oxidant*_ corresponds to the pseudo-first-order rate constant (*k*) of the combined process, while *k*_*US*_ and *k*_*Oxidant*_ represent the *k* values for the individual processes: sonolysis, and direct oxidation, respectively. A value of *S* equal to 1 implies that the combination has an additive effect; if *S* is lower than 1, the combination has an antagonistic effect. Meanwhile, *S* greater than 1 denotes a synergistic effect. The kinetic constants were calculated according to Eq. 2, using a pseudo-first-order kinetic model.

2 $$ln\left(\frac{C}{{C}_{0}}\right)=-kt$$

Before the degradation experiments in SW, HWW, and RU, an initial characterization of the real matrices was carried out by measuring pH, total organic carbon (TOC), and conductivity. The pH was measured using a Mettler Toledo Seven Compact™ pH Meter. The TOC was measured by catalytic combustion in a Shimadzu TOC-L analyzer. The conductivity was determined using a SI Analytics Conductivity Meter Lab 945.

In all experiments, the initial solution pH was not adjusted or buffered, and it was determined by the matrix composition (i.e., the pharmaceutical, the oxidant, and other matrix components, in the case of actual waters evaluation). This considering that at the experimental conditions: (i) HO^•﻿^ radicals sonogeneration is not pH-dependent (Villaroel et al. 2014), (ii) The neutral ACE structure (pKa ~ 9.4) is not affected by the pH variations (2.63–7.41, according to Table S3). Aliquots (1200 μL) were taken at different intervals (0, 10, 20, 40, and 60 min). The samples were used to determine the evolution of oxidants (H_2_O_2_, H_2_O_2_ + PDS, or H_2_O_2_ + PMS) and the removal of ACE. H_2_O_2_ and H_2_O_2_ + PMS evolution was determined by the iodometric method using KI and AHM (Serna-Galvis et al. 2015; Liang and He 2018); while H_2_O_2_ + PDS evolution was monitored by the iodometric method using KI and NaHCO_3_ (Liang et al. 2008), with a Mettler Toledo UV5 Spectrophotometer in all of the cases (see details of the iodometric methods in Text S3).

The removal of ACE was followed using a chromatographic system HPLC-Waters, which consisted of a 1525 binary HPLC pump and a 2487 dual λ absorbance detector, and the software Breeze for data collection. Separation was performed using a LiChrospher® 100 RP-18 (5 µm) column. For the measurement, the samples were introduced through a Rheodyne injector valve with a 20 μL loop and were analyzed using as mobile phase a mixture of MeCN: Milli-Q water 25:75 (% v/v), a flow in an isocratic mode of 0.7 mL min^–1^, and a detection wavelength of 243 nm, during a runtime of 7 min (Retention time: 4.7 min).

## Results and discussion

### Effect of the ultrasonic frequency

**Fig. 1:**
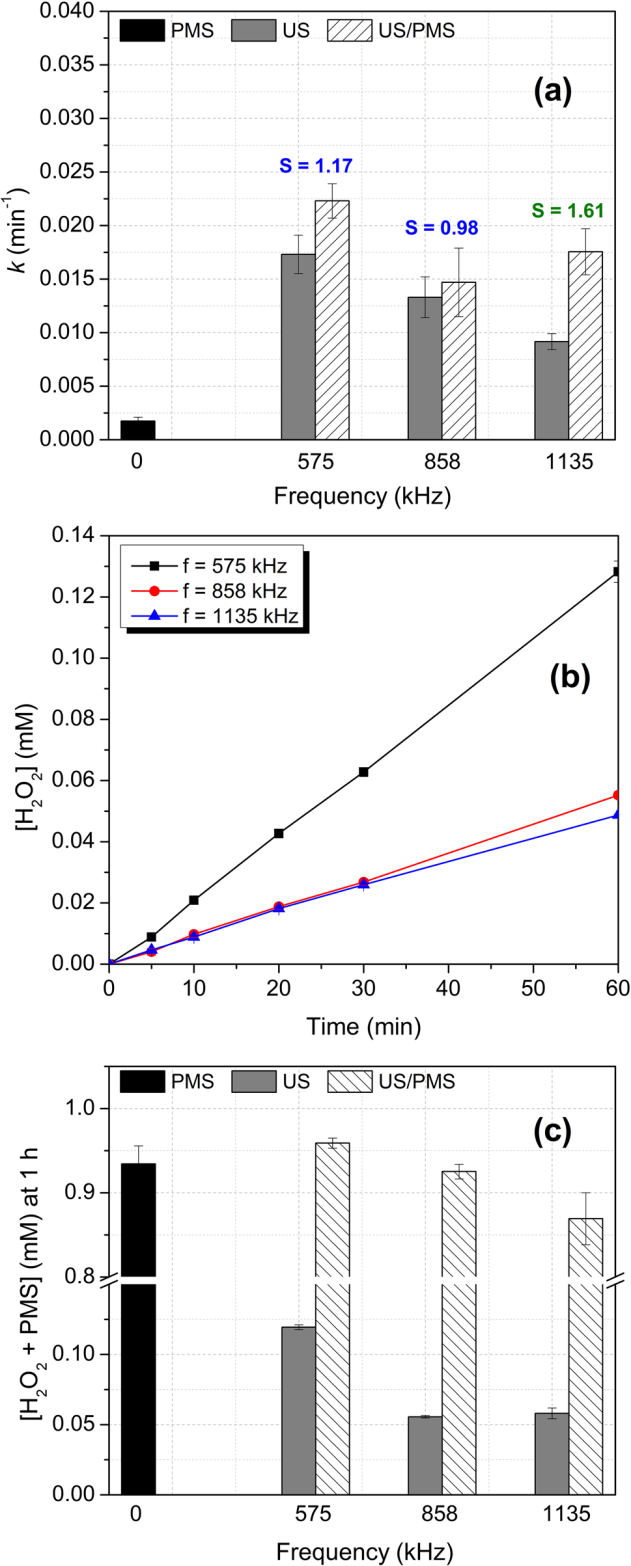
Effect of the ultrasonic frequency. Treatment of ACE by US, direct oxidation with PMS, and the US/PMS system after 1 h of treatment at different frequencies: (**a**) Graph of *k vs*. frequency with the calculated *S* values, (**b**) H_2_O_2_ accumulation during sonication in the absence of ACE, (**c**) oxidants (H_2_O_2_ + PMS) accumulation in the treatment of ACE. Conditions: [ACE]: 40 µM, [PMS]: 1 mM, V: 360 mL, matrix: distilled water, pH_initial_: 5.86 (ACE), 3.18 (ACE + PMS), frequencies of 575, 858, and 1135 kHz, with power densities of 84.14, 87.07, and 85.81 W L^–1^, respectively

The effect of frequency on ACE removal by the US/Oxidant system was initially tested. For this, PMS was selected as the model oxidant. To guarantee the accurate study of the frequency effect, the experiments were carried out at close values of actual acoustic power densities (Text S2). Therefore, for the tested frequencies the actual power densities were 84.14, 87.07, and 85.81 W L^–1^ (for 575, 858, and 1135 kHz, respectively). Figure 1 presents the kinetic constant (*k*) in the ACE treatment by US/PMS at the different ultrasonic frequencies, and the corresponding control experiments of sonolysis and direct oxidation by PMS (1 mM). Figure 1a also presents the synergy values (*S*) for the combined process (detailed graphs of C/C_0_ *vs.* time are shown in Fig. S3).

Figure 1a shows that the degradation of ACE is kinetically more favored at 575 kHz in both cases: in the ultrasound process acting alone and in the combined system (US/PMS). As the frequency increases the size of the cavitation bubbles decreases and the collapse time is shorter, which reduces the extent of radical formation (Lim et al. 2011). In fact, there is a range of frequencies in which bubble size and cavitational events are more prone to the formation of HO^•^. That range has been reported to be between 200–600 kHz (Kang et al. 1999; Torres et al. 2008; Torres-Palma and Serna-Galvis 2018), and consequently, the contaminants are degraded faster at such frequencies. The results of degradation are consistent with the sonolytic experiments carried out in distilled water without the contaminant at different frequencies (Fig. 1b). The production of H_2_O_2_ gives an indirect measurement of HO^•^ formation since it is generated by the combination of hydroxyl radicals (Wang and Zhou 2016; Ferkous et al. 2017).

Figure 1a also depicts the degradation of ACE by PMS alone. By direct oxidation, ~ 12% of the pollutant is degraded after 1 h of treatment. This was related to the redox potential of PMS (E°: 1.82 V) that leads to the oxidation of organic compounds directly (Lee et al. 2021). On the other hand, the results in Fig. 1a show that although the kinetics were favored at 575 kHz followed by 858 kHz, the combination of US with the oxidant lead, in both cases, to an additive effect (S ~ 1). On the contrary, a synergistic effect was observed at the highest studied frequency (i.e., 1135 kHz). These results are in agreement with some reports where the synergistic effects for the US/Oxidant system occur at high frequencies (> 1000 kHz) (Lee et al. 2021). According to our result, kinetics is determined by the intrinsic capability of a system to degrade the target compound (as occurred at 575 and 858 kHz), whereas the synergy depends on the improvement of an inefficient process by the combination with another one (as observed at 1135 kHz).

The higher synergy at 1135 kHz could be due to a decrease in the efficiency in the US alone at this frequency, and also, to the fact that an increase in the frequency promotes an increase in the population of bubbles resulting in a greater number of cavitation events (Torres-Palma and Serna-Galvis 2018). Although these events are not so efficient to generate HO^•^ from water, they could increase the kinetic and mechanical energy in the solution, being able to activate PMS. Thus, the breakdown of PMS produces extra radicals that improve ACE degradation in the system in a synergistic way. This is consistent with oxidant monitoring in ACE degradation (Fig. 1c) where the PMS consumption is higher at 1135 kHz. Therefore, the addition of oxidants could be advantageous at such high frequency since various activation modes take place simultaneously: attack of radicals coming from water sonication, direct pollutant oxidation with PMS, and activation of the oxidant by ultrasonic action.

We should remark that the best synergistic combination does not always imply the highest removal effectiveness. Combined systems can be synergistic at one frequency (e.g., 1135 kHz), but this does not mean they are the most efficient kinetically compared with the same combination at other frequencies (e.g., 575 kHz, Fig. 1a). Furthermore, our results showed that adding oxidants at intermediate frequencies (e.g., 575 kHz), where the sonochemical system alone works well, does not result in a significant improvement. Indeed, the increase in degradation efficiency is due to additive effects between the US alone and the direct action of PMS. Thereby, the combination of the US with PMS is more convenient if the individual systems have low degradation efficiencies, and they can be significantly improved by their combination; but when US or PMS alone works well, it is very difficult to obtain synergistic effects from such combination.

### Effect of the type of oxidant

**Fig. 2:**
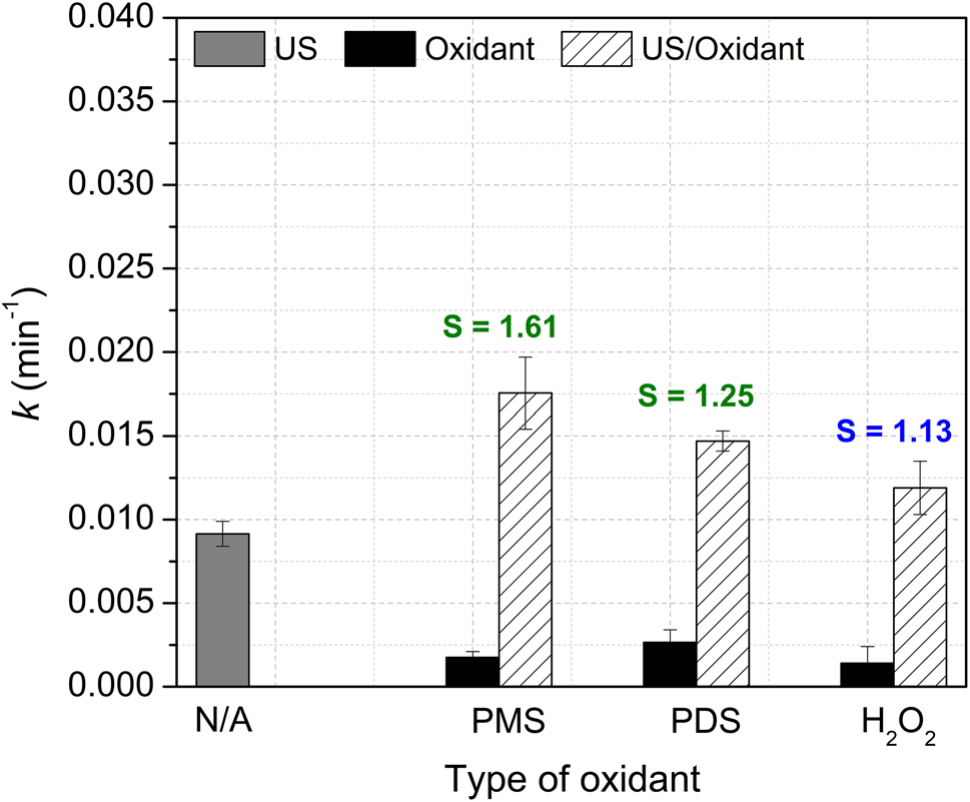
Effect of the type of oxidant. *k* values in the treatment of ACE by US, direct oxidation, and the US/Oxidant system at 1135 kHz, including the calculated *S* values. Conditions: [ACE]: 40 µM, [PMS, PDS, and added H_2_O_2_]: 1 mM, V: 360 mL, matrix: DW, pH_initial_: 5.86 (ACE), 3.18 (ACE + PMS), 5.72 (ACE + PDS), 5.77 (ACE + H_2_O_2_), frequency: 1135 kHz, power density: 85.81 W L^–1^

The results in “Effect of the ultrasonic frequency” section indicated that the research on the synergy promoted by the addition of oxidants should be focused on the US system which is not very efficient for the degradation of pollutants. Therefore, the frequency of 1135 kHz was selected to evaluate the effect of the type of oxidant in the combined system, and the combinations of high-frequency ultrasound with H_2_O_2_, PMS, or PDS were compared. ACE (40 µM) was treated by the US/Oxidant systems, and the direct individual action of these three oxidants (at 1 mM) was also measured. Results for the *k* values in the target pollutant treatment are shown in Fig. 2 (detailed graphs of C/C_0_ *vs.* time and the oxidant accumulation (H_2_O_2_ + PMS or H_2_O_2_ + PDS) can be found in Fig. S4).

Figure 2 shows that the results for both parameters: degradation kinetics and the synergy for the combined system followed the order US/PMS > US/PDS > US/H_2_O_2_, with *k* values of 1.8 × 10^–2^, 1.5 × 10^–2^, and 1.2 × 10^–2^ min^–1^, respectively. These values were higher compared to the direct action of oxidants (1.8 × 10^–3^, 2.7 × 10^–3^, and 1.4 × 10^–3^ min^–1^ using PMS, PDS, and H_2_O_2_, respectively) or the *k* value obtained by the US alone (9.2 × 10^–3^ min^–1^). Consequently, the systems were synergistic by combining US with PMS and PDS and approximately additive using H_2_O_2_.

Several physicochemical properties of the oxidants (PMS, PDS, and H_2_O_2_) have been used to explain the different efficiencies obtained in advanced oxidation processes for the removal of pollutants in the presence of such substances (Guerra-Rodríguez et al. 2018; Lee et al. 2020, 2021; Xia et al. 2020; Zhu et al. 2021). Thus, the oxidation potentials (E°, Table 1, entry i), the structural features (Table 1, entry ii), the lengths and dissociation energies of the O–O bonds (Table 1, entries iii and iv, respectively), as well as properties of their corresponding radical species (oxidation potentials (E°) and half-life times (t_1/2_); Table 1, entries v and vi, respectively) have been considered. However, properties such as the E° of the starting oxidants, and the lengths and dissociation energies of the O–O bonds are very similar for the three oxidants and cannot explain the found results (US/PMS > US/PDS > US/H_2_O_2_).

**Table 1:**
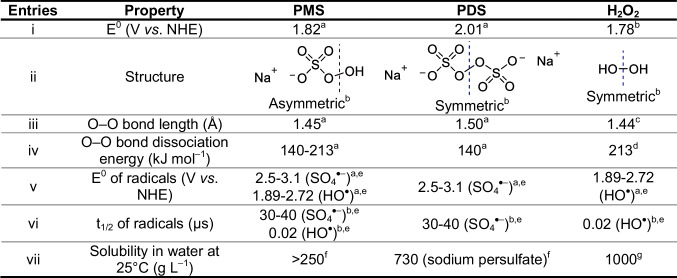
Molecular structures and some physicochemical properties of the oxidants and their corresponding radical species

References: ^a^ (Lee et al. 2021), ^b^ (Wang et al. 2021), ^c^ (Bach and Schlegel 2020), ^d^ (Pang et al. 2011), ^e^ (Bahrami et al. 2018), ^f^ (Guerra-Rodríguez et al. 2018) ^g^ PubChem (pubchem.ncbi.nlm.nih.gov)

On the other hand, cavitation events lead to HO^•^ formation intrinsically (Eqs. 3–7) (Minero et al. 2005; Eren 2012; Serna-Galvis et al. 2016), and the cleavage of the added oxidants can form extra radical species necessary to improve the degradation efficiency of the US/oxidant combination. For instance, HO^•^ and SO_4_^•–^ can be produced from PMS (Eq. 8), whereas only SO_4_^•–^ radicals can be produced from PDS (Eq. 9), and only HO^•^ radicals can be generated from H_2_O_2_ (Eq. 10) (Lee et al. 2021; Wang et al. 2021). Also, the sonogenerated radicals can promote the activation of the oxidants (Eqs. 11–18) (Lim et al. 2014; Wang and Zhou 2016; Lee et al. 2021; Kiejza et al. 2021).

3 $${\mathrm{H}}_{2}\mathrm{O}\,+ \,)))\to {\mathrm{H}}^{\bullet }\,+\,{\mathrm{HO}}^{\bullet }$$

4 $${\mathrm{O}}_{2}\, +\, )))\to {2\mathrm{O}}^{\bullet }$$

5 $${\mathrm{H}}_{2}\mathrm{O}\,+\,{\mathrm{O}}^{\bullet }\to {2\mathrm{HO}}^{\bullet }$$

6 $${\mathrm{H}}_{2}\mathrm{O}\,+\,{\mathrm{H}}^{\bullet }\to {\mathrm{HO}}^{\bullet }\,+\,{\mathrm{H}}_{2}$$

7 $${\mathrm{O}}_{2}\,+\,{\mathrm{H}}^{\bullet }\to {\mathrm{O}}^{\bullet }\,+\,{\mathrm{HO}}^{\bullet }$$

8 $${{\mathrm{HSO}}_{5}}^{-}\,+\,)))\to {{\mathrm{SO}}_{4}}^{\bullet -}\,+\,{\mathrm{HO}}^{\bullet }$$

9 $${\mathrm{S}}_{2}{\mathrm{O}}_{8}^{2-}\,+\,)))\to {{2\mathrm{SO}}_{4}}^{\bullet -}$$

10 $${\mathrm{H}}_{2}{\mathrm{O}}_{2}\,+\,)))\to {2\mathrm{HO}}^{\bullet }$$

11 $${\mathrm{H}}_{2}{\mathrm{O}}_{2}\,+\,{{\mathrm{HO}}_{2}}^{\bullet }\to {\mathrm{H}}_{2}\mathrm{O}\,+\,{\mathrm{HO}}^{\bullet }\,+\,{\mathrm{O}}_{2}$$

12 $${{\mathrm{HSO}}_{5}}^{-}\,+\,{\mathrm{H}}^{\bullet }\to {{\mathrm{SO}}_{4}}^{\bullet -}\,+\,{\mathrm{H}}_{2}\mathrm{O}$$

13 $${{\mathrm{HSO}}_{5}}^{-}\,+\,{{\mathrm{HO}}_{2}}^{\bullet }\to {{\mathrm{SO}}_{4}}^{\bullet -}\,+\,{\mathrm{H}}_{2}\mathrm{O}\,+\,{\mathrm{O}}_{2}$$

14 $${{\mathrm{HSO}}_{5}}^{-}\,+\,{\mathrm{HO}}^{\bullet }\to {{\mathrm{SO}}_{5}}^{\bullet -}\,+\,{\mathrm{H}}_{2}\mathrm{O}$$

15 $${\mathrm{S}}_{2}{{\mathrm{O}}_{8}}^{2-}\,+\,{\mathrm{H}}^{\bullet }\to {{\mathrm{SO}}_{4}}^{\bullet -}\,+\,{{\mathrm{HSO}}_{4}}^{-}$$

16 $${\mathrm{S}}_{2}{{\mathrm{O}}_{8}}^{2-}\,+\,{{\mathrm{HO}}_{2}}^{\bullet }\to {{2\mathrm{SO}}_{4}}^{\bullet -}\,+\,{{\mathrm{O}}_{2}}^{\bullet -}\,+\,{\mathrm{H}}^{+}$$

17 $${\mathrm{S}}_{2}{{\mathrm{O}}_{8}}^{2-}\,+\,{\mathrm{HO}}^{\bullet }\to {{\mathrm{SO}}_{4}}^{\bullet -}\,+\,{{\mathrm{HSO}}_{4}}^{-}\,+\,1/{2\mathrm{O}}_{2}$$

18 $${{\mathrm{SO}}_{4}}^{\bullet -}+{\mathrm{H}}_{2}\mathrm{O}/{\mathrm{OH}}^{-}\to {{\mathrm{HSO}}_{4}}^{-}/{{\mathrm{SO}}_{4}}^{2-}+{\mathrm{HO}}^{\bullet }$$

Sulfate radicals, which can be sonochemically generated in the presence of PMS or PDS (Eq. 8–9), have a redox potential similar to hydroxyl radicals but a longer half-life time (t_1/2_) (Table 1, entries v and vi), which allows sulfate radicals to have more chance to interact with the organic pollutants and degrade them. This explains the better efficiency obtained by the US/PMS and US/PDS processes compared to the US/H_2_O_2_ system, but it does not allow to explain why the US/PMS system is more efficient than the US/PDS system, mainly because, in both systems, many other reactions take place generating extra HO^•^ and SO_4_^•–^ radicals, together with other reactive species (Eqs. 3–18).

Interestingly, it can be seen (Table 1, entry ii) that PMS has an asymmetric structure, while PDS and H_2_O_2_ have a symmetric peroxide bond. SO_3_^–^ is an electro-withdrawing group. Therefore, in PMS, the O–O bond electron density leans toward the SO_3_^–^, leaving the O on the H side with a positive charge density (Zhu et al. 2021). Therefore, the cleavage of the O–O bond in PMS can occur more easily than in symmetric structures such as PDS or H_2_O_2_ (Wang et al. 2021), which explains the best results observed for the US/PMS system.

Finally, the better performance found in the US/PMS system compared to the US/PDS and the US/H_2_O_2_ systems may be also related to the hydrophobicity/hydrophilicity of the oxidants, which is a determinant property for oxidant activation. In fact, hydrophobicity is associated with the proximity of the oxidants to the interface between the cavity and the solution bulk where their activation towards radical formation occurs (Seymour and Gupta 1997; Nanzai et al. 2008; Wei et al. 2016). The solubility was selected as an indicator of the degree of hydrophilicity (Table 1, entry vii). As seen, solubility increases in the order PMS < PDS < H_2_O_2_. Then, PMS is more hydrophobic and it will migrate more easily toward the bubble interface, where it can be activated to the corresponding radicals. Thereby, these differences in chemical structures and hydrophilicity explain the results in kinetics and synergies observed in Fig. 2.

### Effect of initial concentration of ACE

**Fig. 3:**
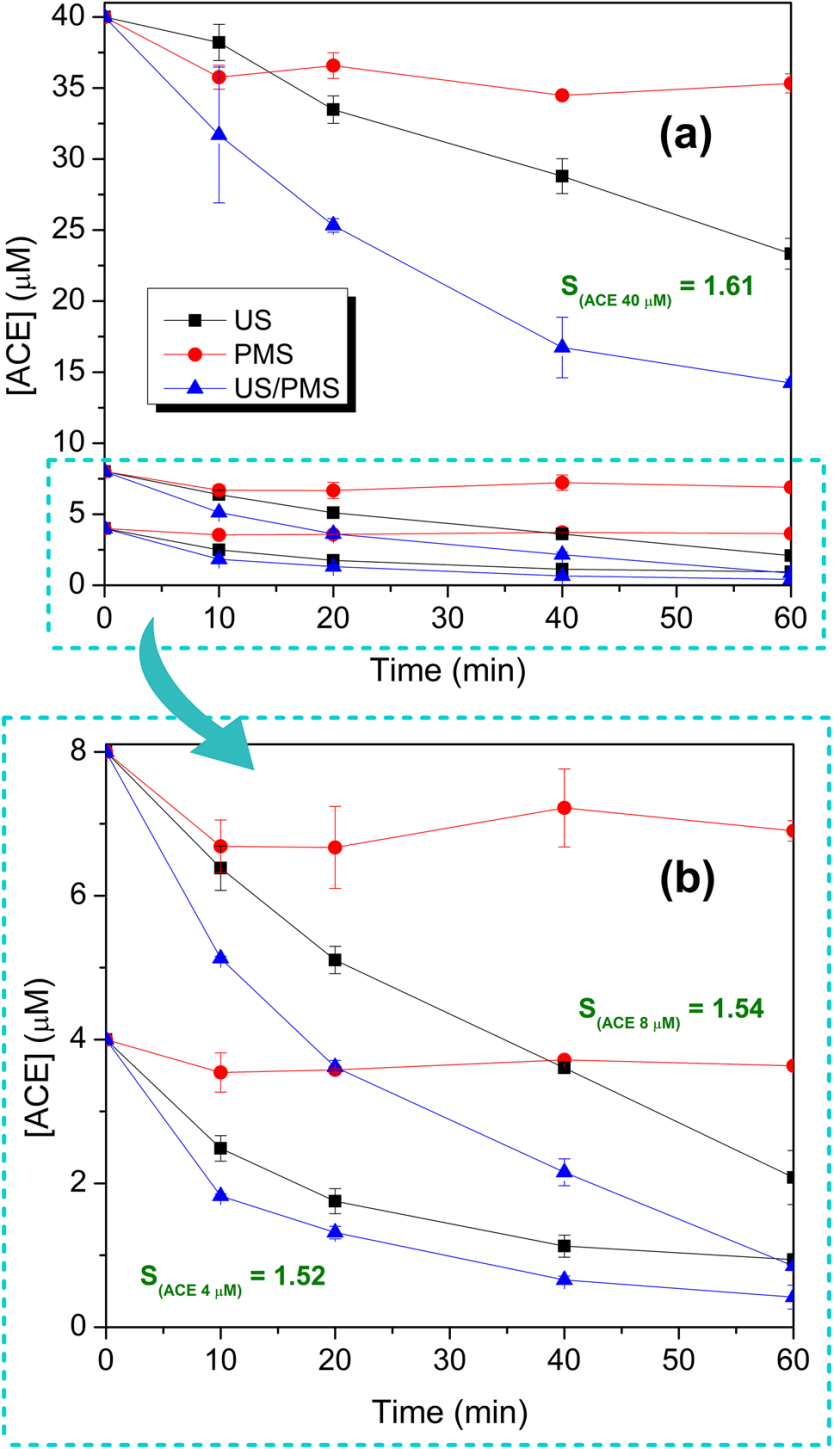
Effect of initial ACE concentration. Treatment of ACE by US, direct oxidation, and the US/PMS system at 1135 kHz: (**a**, **b**) Concentration of ACE *vs.* time with the calculated *S* values. Conditions: [ACE]: 4, 8, and 40 µM, [PMS]: 1 mM, V: 360 mL, matrix: DW, pH_initial_: 5.77–5.86 (ACE 4–40 µM), 3.18 (ACE 4–40 µM + PMS), frequency: 1135 kHz, power density: 85.81 W L^–1^

To study the effect of the initial concentration of the pollutant, degradation experiments were performed using the best US(1135 kHz)/PMS(1.0 mM) combination and three different concentrations of ACE (4, 8, and 40 µM). Control experiments of sonolysis and direct oxidation were also performed. Results are shown in Fig. 3 (detailed graphs of C/C_0_ *vs.* degradation time and the oxidant accumulation (H_2_O_2_ + PMS) are shown in Fig. S5).

It can be seen that after 1 h of treatment, the US system eliminated 3.06, 5.92, and 16.67 µM from initial ACE concentrations of 4, 8, and 40 µM, respectively. Direct oxidation by PMS removed 0.36, 1.10, and 4.68 µM of the initial concentrations of 4, 8, and 40 µM of ACE, respectively (Fig. 3a and b). Meanwhile, for the combined US/PMS system, it was possible to remove 3.58, 7.15, and 25.76 µM from the initial concentrations of 4, 8, and 40 µM of ACE, respectively. This means that the sonochemical process, the direct oxidation, and the US/PMS system are more effective at the highest concentration of ACE studied since they can degrade a greater amount of moles of the target contaminant. Additionally, although the processes with the different concentrations of ACE were synergistic, the highest synergistic effect of the US/PMS combination occurs at the highest concentration of ACE. This may be because a higher concentration of the contaminant increases the number of dispersed molecules in the solution; therefore, the probability that these molecules approach the cavitation bubbles and the sites with the highest concentration of radicals (HO^•^ and SO_4_^•–^) is greater, thus favoring degradation. However, it has been reported that concentrations of the contaminant above a threshold value can saturate the interfacial region of cavities, decreasing the degradation (Panda et al. 2020).

### Effect of oxidant concentration

**Fig. 4:**
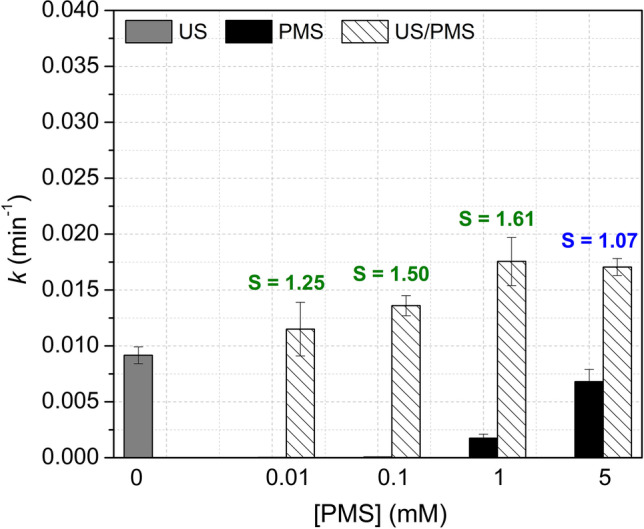
Effect of oxidant concentration. *k* values in the treatment of ACE by US, direct oxidation, and the US/PMS system after 1 h of treatment at 1135 kHz with the calculated *S* values. Conditions: [ACE]: 40 µM, [PMS]: 0.01, 0.1, 1, and 5 mM, V: 360 mL, matrix: DW, pH_initial_: 5.86 (ACE), 5.25 (ACE + PMS 0.01 mM), 4.16 (ACE + PMS 0.1 mM) 3.18 (ACE + PMS 1 mM), 2.69 (ACE + PMS 5 mM), frequency: 1135 kHz, power density: 85.81 W L^–1^

To determine the effect of the oxidant concentration on the synergistic effects of the US(1135 kHz)/PMS combination, the kinetics in the ACE removal (40 µM) using several PMS concentrations (0.01, 0.1, 1, and 5 mM) was evaluated. Direct oxidation was also evaluated at the selected PMS concentrations. Results are shown in Fig. 4 (detailed graphs of C/C_0_ *vs.* degradation time and the oxidant accumulation (H_2_O_2_ + PMS) are shown in Fig. S6).

Figure 4 shows that as the PMS concentration increased, the degradation kinetics augmented in the direct oxidation (PMS) and the US/PMS systems. However, the synergistic effect was observed at PMS concentrations of 0.01, 0.1, and 1 mM, being 1, and 0.1 mM PMS the best synergistic options (*S* = 1.61 and 1.50, respectively). When PMS concentration is very low (0.01 mM), the synergistic effect of the US/PMS combination decreases because PMS at low concentration tends to disperse within the solution, making it more difficult for PMS molecules to approach the cavity-solution bulk interface, which is critical to favor the formation of radicals. On the contrary, when the concentration of PMS is increased to 5 mM, the effect of the combination decreases and becomes additive. This is because PMS at high concentrations, although highly efficient in the direct oxidation of ACE, can favor undesired reactions. In fact, at very high PMS concentrations, this oxidant and the species generated from this can also act as a scavenger of the generated radicals (Eqs. 19–24, (Wang and Zhou 2016; Xu et al. 2020a; Wang et al. 2021)) or can lead to recombination reactions (Eqs. 25–27, (Wang and Zhou 2016; Ferkous et al. 2017; Cui et al. 2021b)), which decreases both the efficiency and the synergy of the ACE degradation.

19 $${\mathrm{H}}_{2}{\mathrm{O}}_{2}\,+\,{\mathrm{HO}}^{\bullet }\to {\mathrm{H}}_{2}\mathrm{O}+{{\mathrm{HO}}_{2}}^{\bullet }$$

20 $${{\mathrm{HSO}}_{5}}^{-}\,+\,{\mathrm{HO}}^{\bullet }\to {{\mathrm{SO}}_{5}}^{\bullet -}\,+\,{\mathrm{H}}_{2}\mathrm{O}$$

21 $${{\mathrm{HSO}}_{5}}^{-}\,+\,{{\mathrm{SO}}_{4}}^{\bullet -}\to {{\mathrm{HSO}}_{4}}^{-}\,+\,{{\mathrm{SO}}_{5}}^{\bullet -}$$

22 $${{2\mathrm{SO}}_{5}}^{\bullet -}\to {\mathrm{S}}_{2}{{\mathrm{O}}_{8}}^{2-}\,+\,{\mathrm{O}}_{2}$$

23 $${\mathrm{S}}_{2}{{\mathrm{O}}_{8}}^{2-}\,+\,{\mathrm{HO}}^{\bullet }\to {\mathrm{HO}}^{-}\,+\,{\mathrm{S}}_{2}{{\mathrm{O}}_{8}}^{\bullet -}$$

24 $${\mathrm{S}}_{2}{{\mathrm{O}}_{8}}^{2-}\,+\,{{\mathrm{SO}}_{4}}^{\bullet -}\to {{\mathrm{SO}}_{4}}^{2-}\,+\,{\mathrm{S}}_{2}{{\mathrm{O}}_{8}}^{\bullet -}$$

25 $$\begin{array}{cc}{2\mathrm{HO}}^{\bullet }\to {\mathrm{H}}_{2}{\mathrm{O}}_{2}& k=5.5\times {10}^{9}\end{array} {\mathrm{M}}^{-1} {\mathrm{s}}^{-1}$$

26 $$\begin{array}{cc}{\mathrm{HO}}^{\bullet }\,+\,{{\mathrm{SO}}_{4}}^{\bullet -}\to {{\mathrm{HSO}}_{5}}^{-}& k=1.0\times {10}^{10}\end{array} {\mathrm{M}}^{-1}{\mathrm{s}}^{-1}$$

27 $$\begin{array}{cc}{{2\mathrm{SO}}_{4}}^{\bullet -}\to {\mathrm{S}}_{2}{{\mathrm{O}}_{8}}^{2-}& k=3.1\times {10}^{8} {\mathrm{M}}^{-1}{\mathrm{s}}^{-1}\end{array}$$

The above results indicated that the addition of PMS to the US system requires a suitable amount of the oxidant, which enhances the degradation and synergy. At high PMS concentrations (e.g., 5 mM), its consumption was elevated but scavenger effects, as mentioned previously, seem to dominate.

### Evaluation of the US/PMS system in real aqueous matrices

**Fig. 5:**
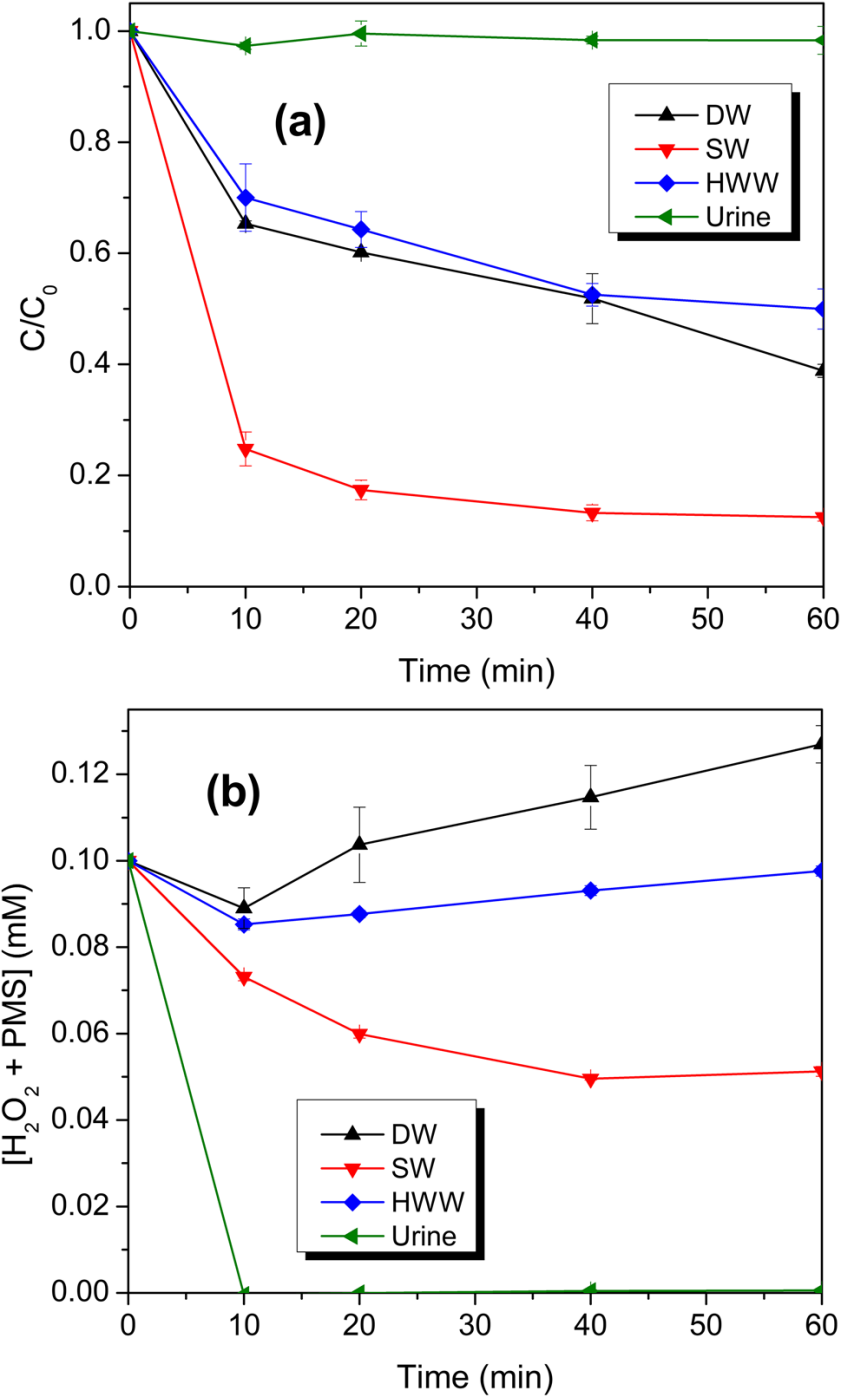
Degradation of ACE by the US/PMS system in different matrixes: (**a**) C/C_0_ *vs.* degradation time, (**b**) Oxidant (H_2_O_2_ + PMS) accumulation. Conditions: [ACE]: 40 µM, [PMS]: 0.1 mM, V: 360 mL, matrixes: DW (black curves), SW (red curves), HWW (blue curves), and urine (green curves), pH_initial_: 4.16 (ACE + PMS in DW), 7.53 (ACE + PMS in SW), 7.58 (ACE + PMS in HWW), 6.84 (ACE + PMS in urine), frequency: 1135 kHz, power density: 85.81 W L^–1^

The treatment of ACE in three relevant matrices (SW, HWW, and urine, see Table 2) was considered. Based on the previous results, a PMS concentration of 0.1 mM was chosen for the experiments. This considering that although 1 mM PMS led to the highest synergy, 10 times less PMS led to a very similar synergy value. The target pollutant was spiked into these aqueous media to evaluate the degradation. The results for the ACE treatments in the complex matrices, using the US/PMS system, are shown in Fig. 5.

**Table 2:** Characteristics of the real matrices*

Real matrices	pH	TOC (mg L^–1^)	Conductivity (μS cm^–1^)
Distilled water (DW)	5.97	0.24	1.5
Seawater (SW)	7.77	2.84	48900
Hospital wastewater (HWW)	7.63	6.46	438
Urine	6.92	2777	9460

*More information on the HWW of the city of Tumaco-Colombia and a more detailed description of the matrix composition can be consulted in references (Serna-Galvis et al. 2019c, 2022)

As seen in Fig. 5a, compared to the treatment of ACE in distilled water (DW), the HWW slightly inhibited the degradation of the pollutants. In contrast, the urine components inhibited the degradation completely. Remarkably, the removal of the pharmaceutical in seawater was significantly intensified. The few effects in HWW are explained considering that it has a low amount of organic matter of 6.46 mg L^–1^ (Table 2), which competes moderately with ACE (3.84 mg L^–1^ in TOC) for the degrading species. This is also evidenced in the monitoring of oxidants (Fig. 5b), wherein in the HWW, there is moderate consumption of the oxidants. On the contrary, the urine has a very high organic load (Table 2), so the degradation of ACE was inhibited entirely because the added and sono-generated oxidants are easily consumed (in only 10 min) in the oxidation of the non-recalcitrant organic matter present. Interestingly, the degradation of ACE in SW was significantly intensified; this is due not only to the fact that the organic load in seawater is very low (TOC: 2.84 mg L^–1^) but also to the fact that there is a high content of inorganic species such as chloride ions that could promote the so-called "salting-out" effect, pushing the target pollutant close to the cavitation bubble, favoring its sono-degradation (Serna-Galvis et al. 2015). Furthermore, it is reported that at a very high concentration of chloride ions (as present in the SW matrix), PMS can react with this anion, producing HOCl (Eq. 28) (Lou et al. 2013; Zhou et al. 2018; Liu et al. 2020), which can also attack ACE (Flores-Terreros et al. 2022), thus enhancing the pollutant degradation.

Although it is reported that the reaction of HO^•^ and SO_4_^•–^ with chloride ions generates other species such as Cl_2_^•–^, which can degrade organic pollutants (Eqs. 29–32), the quenching of HO^•^ and SO_4_^•–^ by chloride ions usually leads to decreased degradation efficiency (Li et al. 2013; Lu et al. 2019). These effects of the matrix are also supported by the high oxidant consumption in SW, as seen in Fig. 5b.

28 $${\mathrm{Cl}}^{-}\,+\,{{\mathrm{HSO}}_{5}}^{-}\to \mathrm{HOCl}\,+\,{{\mathrm{SO}}_{4}}^{2-}$$

29 $${\mathrm{Cl}}^{-}\,+\,{\mathrm{HO}}^{\bullet }\to {\mathrm{HOCl}}^{\bullet -}$$

30 $${\mathrm{HOCl}}^{\bullet -}\,+\,{\mathrm{Cl}}^{-}\to {{\mathrm{Cl}}_{2}}^{\bullet -}\,+\,{\mathrm{HO}}^{-}$$

31 $${\mathrm{Cl}}^{-}\,+\,{{\mathrm{SO}}_{4}}^{\bullet -}\to {\mathrm{Cl}}^{\bullet }\,+\,{{\mathrm{SO}}_{4}}^{2-}$$

32 $${\mathrm{Cl}}^{\bullet }\,+\,{\mathrm{Cl}}^{-}\to {{\mathrm{Cl}}_{2}}^{\bullet -}$$

It has been also informed that the presence of inorganic species also decreases the solubility of the gas in solution and reduces the amount of large degassed bubbles, which would otherwise attenuate the acoustic wave. Due to this, the sonochemical yield decreases with increasing NaCl concentration, but the sonoluminescence (SL) intensity, which is produced by the collapse of the bubbles, increases (Pflieger et al. 2019). The SL phenomenon can also lead to a photochemical and thermal activation of the PMS, so this physical effect caused by the presence of NaCl would be added to the increase in the degradation of ACE in SW.

It is important to mention that, for the experiments in the different matrices, the measurement of the final TOC resulted in similar values to the initial TOC shown in Table 2 (data not shown), with which it is concluded that the US/PMS combination similar to the independent US, it is not efficient for mineralization (conversion of pollutants and degradation by-products to CO_2_ and water).

## Conclusions

This study showed that the combination of ultrasound with oxidants has contrasting effects on synergy and kinetics. The synergistic effects of the combination occur at frequencies where the US does not work very well (e.g., 1135 kHz). However, the degradation kinetics were more significant at intermediate frequencies (i.e., 575 kHz). The oxidant that best favored both kinetics and synergy was PMS, which has structural and physicochemical properties that favor its activation by the US into HO^•^ and SO_4_^•–^. Thereby, the US/PMS system was more effective at the highest concentration of ACE studied, and a moderate PMS concentration was most appropriate to favor the synergy. Additionally, the system was highly effective to degrade the model contaminant in seawater or hospital wastewater but inefficient in water matrices with a high organic load, such as real urine. We can highlight that this study contributed to the understanding of strategies to intensify the US technique by activating oxidants, revealing that a synergistic system does not always imply that it is the most kinetically favorable for the degradation of pollutants.

## Supplementary Information

Below is the link to the electronic supplementary material.

Supplementary file1 (DOCX 1.34 MB)

## Data Availability

Data and materials will be available upon request to the authors.
