# Supplementary material for: Systematic study of the synergistic and kinetics effects on the removal of contaminants of emerging concern from water by ultrasound in the presence of diverse oxidants
Source: Environ Sci Pollut Res Int. 2023 Aug 26;32(16):10478–89. doi: 10.1007/s11356-023-29189-y (PMC11996977; doi:10.1007/s11356-023-29189-y)
Supplement: Supplementary file 1 — Supplementary file1 (DOCX 1.34 MB) [file 11356_2023_29189_MOESM1_ESM.docx]

**SUPPORTING INFORMATION**

**Systematic study of the synergistic and kinetics effects on the removal of contaminants of emerging concern from water by ultrasound in the presence of diverse oxidants**

Sandra E. Estrada-Flórez^a^, Efraím A. Serna-Galvis^a,b^, Judy Lee^c^, Ricardo A. Torres-Palma^a,^*

*^a^ Grupo de Investigación en Remediación Ambiental y Biocatálisis (GIRAB), Instituto de Química, Facultad de Ciencias Exactas y Naturales, Universidad de Antioquia UdeA, Calle 70 No. 52-21, Medellín, Colombia*

^b^ *Grupo de Catalizadores y Adsorbentes (CATALAD), Instituto de Química, Facultad de Ciencias Exactas y Naturales, Universidad de Antioquia UdeA, Calle 70 No. 52-21, Medellín, Colombia*

^c^ *School of Chemistry and Chemical Engineering, University of Surrey, Guildford, GU2 7XH, United Kingdom*

*Corresponding author: ricardo.torres@udea.edu.co (R. A. Torres-Palma)

**List of texts**

[Text S1. Selection of the model pollutant 5](#_Toc136950061)

[Text S2. Calorimetric measurements 6](#_Toc136950062)

[Text S3. Brief details on the iodometric methods 9](#_Toc136950063)

**List of Figures**

[Fig. S1. Graphs of T *vs*. time at different ultrasonic frequencies (Adjusted power: 80 W). 6](#_Toc132017488)

[Fig. S2. Schematic diagram of the ultrasonic reactor. 7](#_Toc132017489)

[Fig. S3. Effect of the ultrasonic frequency. Degradation of ACE by US (black curves), direct oxidation (red curves), and the US/PMS system (blue curves) at different frequencies: (a) C/C_0_ *vs.* degradation time, (b) Oxidant accumulation (H_2_O_2_ + PMS).. 10](#_Toc132017490)

[Fig. S4. Effect of the type of oxidant. Degradation of ACE by US (black curves), direct oxidation (red curves), and the US/Oxidant system (blue curves) at 1135 kHz: (a) C/C_0_ *vs.* degradation time, (b) Oxidant accumulation (H_2_O_2_ + PMS or PDS). 11](#_Toc132017491)

[Fig. S5. Effect of initial ACE concentration. Degradation of ACE by US (black curves), direct oxidation (red curves), and the US/PMS system (blue curves) at 1135 kHz: (a) C/C_0_ *vs.* degradation time, (b) Oxidant accumulation (H_2_O_2_ + PMS). 12](#_Toc132017492)

[Fig. S6. Effect of oxidant concentration. Degradation of ACE by US (black curves), direct oxidation (red curves), and the US/PMS system (blue curves) at 1135 kHz: (a) C/C_0_ *vs.* degradation time, (b) Oxidant accumulation (H_2_O_2_ + PMS). 13](#_Toc132017493)

**List of tables**

[Table S1. Physicochemical properties of ACE according to its structure at the initial experimental pH, and the degree of hydrophobicity/hydrophilicity. 5](#_Toc136936083)

[Table S2. Determined PD values at the different frequencies (Adjusted power: 80 W). 6](#_Toc136936084)

[Table S3. Initial and final pHs (after 1 h of treatment) in all the performed experiments. 8](#_Toc136936085)

Text S1. Selection of the model pollutant

Acetaminophen (ACE) is one of the first resources to relieve pain, so self-medication and lack of knowledge about the implications of high consumption of this PhP have made it one of the most consumed worldwide. Therefore, its presence has been detected in waters in various concentrations in different parts of the world, implying that ACE and its metabolites cause negative impacts on aquatic and ecological systems and human health (Fisher and Curry 2019; Phong Vo et al. 2019). Although it is known that an ACE concentration <71 ng L^–1^ makes water sustainable for drinking purposes (Vulliet and Cren-Olivé 2011), the concentration in many countries exceeds this value, and significant efforts must be made to decrease its concentration in different water bodies. Based on the data reported by different articles and reviews, it is estimated that ACE concentration is in the range between 0.04 and 300 µg L^–1^ in influents and effluents of wastewater treatment plants (WWTPs), 0.2 and 220 µg L^–1^ in hospital wastewaters effluents (HWW), <10 µg L^–1^ in surface waters, and <2 µg L^–1^ in groundwater (Zhao et al. 2018; Botero-Coy et al. 2018; Patel et al. 2019; Phong Vo et al. 2019).

ACE was selected in this study because it has intermediate characteristics between hydrophilicity and hydrophobicity. Therefore, it may have intermediate ultrasonic degradation compared to other highly hydrophilic or hydrophobic compounds (See chemical structure and physicochemical properties of ACE in Table S1). The natural pH of ACE in distilled water is 5.86, a value that is below its pKa (9.38-9.46, -OH group), in all experiments, ACE is found in a neutral form, and the addition of oxidants generally causes the pH to decrease, in its neutral form its octanol-water partition coefficient (Log P) is 0.68, making it slightly hydrophilic.

Table S1. Physicochemical properties of ACE according to its structure at the initial experimental pH, and the degree of hydrophobicity/hydrophilicity.

| Natural pH | pKa | Log P^b^ | | TPSA (Å^2^)^b^ | |
| --- | --- | --- | --- | --- | --- |
|  |  | Neutral | Anion | Neutral | Anion |
| 5.86 | 9.38-9.46 (-OH)^a^ | **0.68^c^** | -2.12 | **49.33^c^** | 52.16 |

^a^Data was taken from PubChem ([https://pubchem.ncbi.nlm.nih.gov](https://pubchem.ncbi.nlm.nih.gov/)). ^b^Log P and TPSA values were calculated using molinspiration (<https://www.molinspiration.com/cgi-bin/properties>). ^c^Log P and TPSA values for the ACE species present at the working pH (natural pH and after the addition of oxidants).

Text S2. Calorimetric measurements

Ultrasonic power dissipated per liter of the solution (W L^–1^) was determined at the different frequencies evaluated (575, 858, and 1135 kHz). With this purpose, the power of the equipment was adjusted to 80 W (amplitude 80%), and 360 mL of water were sonicated without a thermostatic bath. The temperature (T) was followed every 15 s for 3 min. The plots of T *vs*. time at each frequency are shown in Fig. S1.


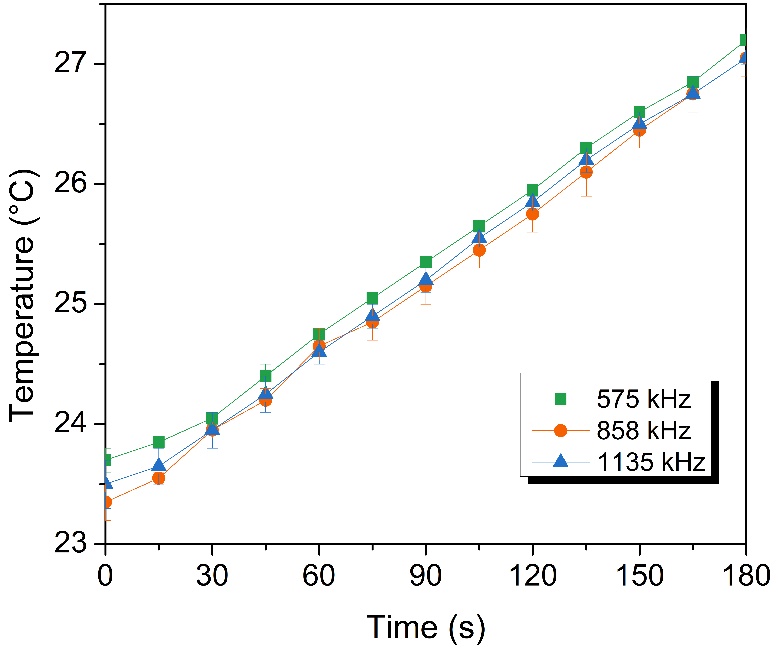


Fig. S1. Graphs of T *vs*. time at different ultrasonic frequencies (Adjusted power: 80 W).

The real power density (PD) has been determined by using Eq. S1 (Kimura et al. 1996).

| $PD=\frac{s\times c\times m}{V}$ | (Eq. S1) |
| --- | --- |

Where *s* is the slope of the graphs of T *vs*. time in °C s^–1^, *c* is the specific heat of water (4.186 J g^–1^ °C^–1^), *m* is the mass of water in g, and *V* is the volume of the irradiated solution. The results of the calorimetric measurements are shown in Table S2.

Table S2. Determined PD values at the different frequencies (Adjusted power: 80 W).

| **Frequency (kHz)** | **Power density (W L^–1^)** | **Error** |
| --- | --- | --- |
| 575 | 84.14 | 0.84 |
| 858 | 87.07 | 1.26 |
| 1135 | 85.81 | 2.09 |


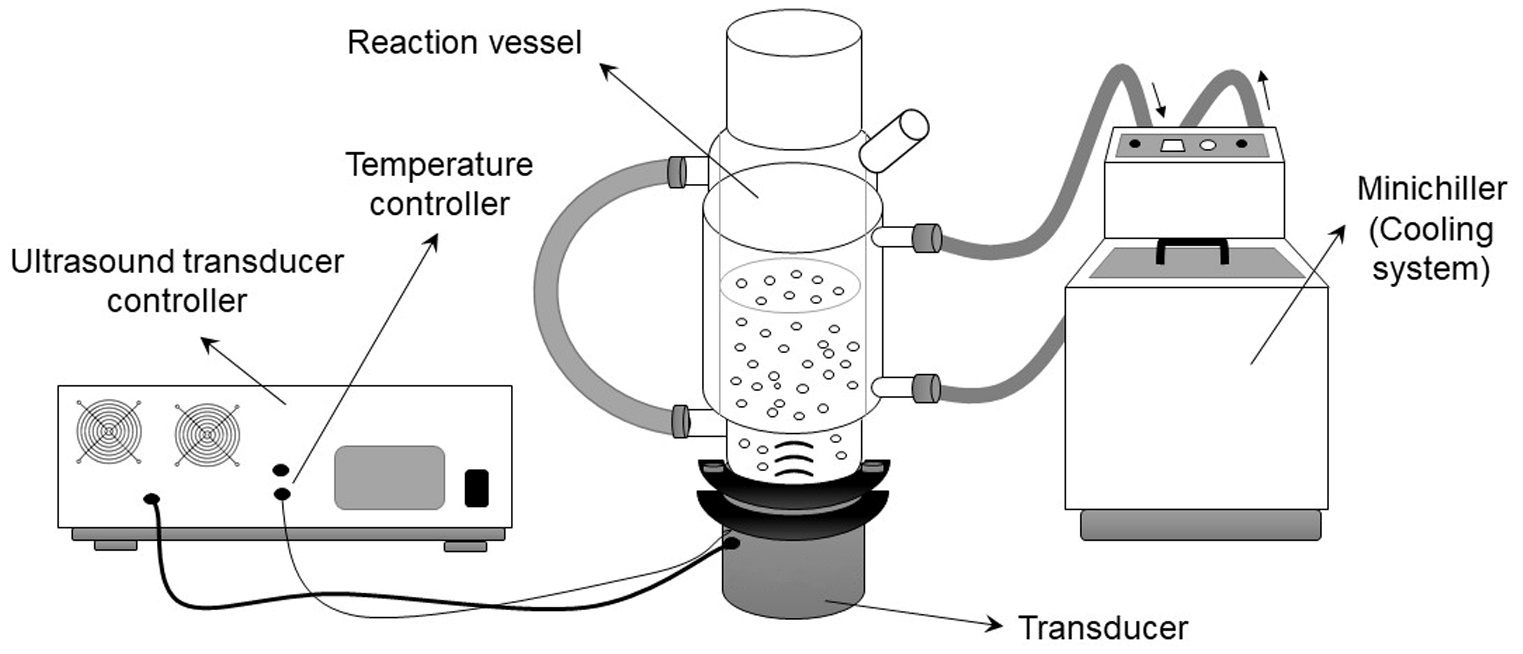


Fig. S2. Schematic diagram of the ultrasonic reactor.

Table S3. Initial and final pHs (after 1 h of treatment) in all the performed experiments.

| **Experiment** | ***f* (kHz)** | **[PhP] (µM)** | **natural pH** | **[Oxidant] (mM)** | **pH_i_ with oxidant** | **pH_f_** |
| --- | --- | --- | --- | --- | --- | --- |
| ACE-PMS | n.a. | 4 | 5.77 | 1 | 3.18 | 3.18 |
| ACE-PMS | n.a. | 8 | 5.75 | 1 | 3.19 | 3.17 |
| ACE-PMS | n.a. | 40 | 5.86 | 0.01 | 5.25 | 5.02 |
| ACE-PMS | n.a. | 40 | 5.86 | 0.1 | 4.16 | 4.16 |
| ACE-PMS | n.a. | 40 | 5.86 | 1 | 3.18 | 3.18 |
| ACE-PMS | n.a. | 40 | 5.86 | 5 | 2.69 | 2.66 |
| ACE-PDS | n.a. | 40 | 5.86 | 1 | 5.72 | 5.63 |
| ACE-H_2_O_2_ | n.a. | 40 | 5.86 | 1 | 5.77 | 5.73 |
| ACE-PMS in SW | n.a. | 40 | 7.75 | 0.1 | 7.53 | 6.97^a^ |
| ACE-PMS in HWW | n.a. | 40 | 7.74 | 0.1 | 7.58 | 7.41 |
| ACE-PMS in urine | n.a. | 40 | 6.87 | 0.1 | 6.84 | 6.89 |
| ACE-US | 1135 | 4 | 5.77 | n.a. | n.a. | 3.98 |
| ACE-US | 1135 | 8 | 5.75 | n.a. | n.a. | 4.01 |
| ACE-US | 575 | 40 | 5.86 | n.a. | n.a. | 3.83 |
| ACE-US | 858 | 40 | 5.86 | n.a. | n.a. | 4.08 |
| ACE-US | 1135 | 40 | 5.86 | n.a. | n.a. | 4.00 |
| ACE-US/PMS | 1135 | 4 | 5.77 | 1 | 3.18 | 3.18 |
| ACE-US/PMS | 1135 | 8 | 5.75 | 1 | 3.19 | 3.17 |
| ACE-US/PMS | 575 | 40 | 5.86 | 1 | 3.18 | 3.09 |
| ACE-US/PMS | 858 | 40 | 5.86 | 1 | 3.18 | 3.11 |
| ACE-US/PMS | 1135 | 40 | 5.86 | 0.01 | 5.25 | 4.01 |
| ACE-US/PMS | 1135 | 40 | 5.86 | 0.1 | 4.16 | 3.77 |
| ACE-US/PMS | 1135 | 40 | 5.86 | 1 | 3.18 | 3.13 |
| ACE-US/PMS | 1135 | 40 | 5.86 | 5 | 2.69 | 2.63 |
| ACE-US/PDS | 1135 | 40 | 5.86 | 1 | 5.72 | 3.95 |
| ACE-US/H_2_O_2_ | 1135 | 40 | 5.86 | 1 | 5.77 | 4.03 |
| ACE-US/PMS in SW | 1135 | 40 | 7.75 | 0.1 | 7.53 | 6.97 |
| ACE-US/PMS in HWW | 1135 | 40 | 7.74 | 0.1 | 7.58 | 7.41 |
| ACE-US/PMS in RU | 1135 | 40 | 6.87 | 0.1 | 6.84 | 6.88 |

^a^ pH_f_ after 2 h of treatment.

Text S3. Brief details on the iodometric methods

The H_2_O_2_ and the sum of oxidants (H_2_O_2_ + PMS) were determined by the iodometric method using potassium iodide (KI) and ammonium heptamolybdate (AHM) (Serna-Galvis et al. 2015; Liang and He 2018); while the sum of H_2_O_2_ + PDS was monitored by the iodometric method using KI and NaHCO_3_ (Liang et al. 2008). Considering that these iodometric methods are not selective, it is difficult to follow the oxidants (HO^•^, H_2_O_2_, SO_4_^•–^, HSO_5_^–^ (PMS), and S_2_O_8_^2–^ (PDS)) independently in the reaction mixture during the treatment by the US/Oxidant system. Instead, these iodometric methods are used for monitoring the oxidants added or produced by recombination of the sono-generated radicals HO^·^ and SO_4_^·–^ (Eqs. S2-S4) (Wang and Zhou 2016; Ferkous et al. 2017).

| 2HO^•^ → H_2_O_2_ | (Eq. S2) |
| --- | --- |
| HO^•^ + SO_4_^•–^ → HSO_5_^–^ | (Eq. S3) |
| 2SO_4_^•–^ → S_2_O_8_^2–^ | (Eq. S4) |

Briefly, in the iodometric method for determination of H_2_O_2_, PMS, and H_2_O_2_ + PMS, 50 µL of 0.01 M AHM, and 1135 µL of 0.1 M KI are added to 600 µL of the sample, which was taken at different intervals during the sonochemical treatment, giving a total volume of 2 mL. The reaction mixture is allowed to react for 5 min and the absorbance at 353 nm is measured.

In the iodometric method to determine PDS, and H_2_O_2_ + PDS, 50 mL of a mixture of 0.6 M KI (5 g) and 0.06 M NaHCO_3_ (0.25 g) was prepared. For the determination, 2 mL of the mixture was allowed to react with 200 µL of the samples taken at different intervals. It was left to react for 15 min and the absorption at 353 nm was measured.

The reactions that take place by these methods are shown in Eqs. S5-S8. The concentration of the oxidants in the samples was calculated by correcting the absorbance with the dilution factor and using the molar absorptivity of the triiodide ion (ɛ = 26400 M^–1^cm^–1^).

| H_2_O_2_ + 2I^–^ → 2HO^–^ + I_2_ | (Eq. S5) |
| --- | --- |
| HSO_5_^–^+ 2I^–^ → SO_4_^2–^ + HO^–^ + I_2_ | (Eq. S6) |
| S_2_O_8_^2–^ + 2I^–^ → 2SO_4_^2–^ + I_2_ | (Eq. S7) |
| I_2_ + I^–^ → I_3_^–^ (Abs at 353 nm) | (Eq. S8) |


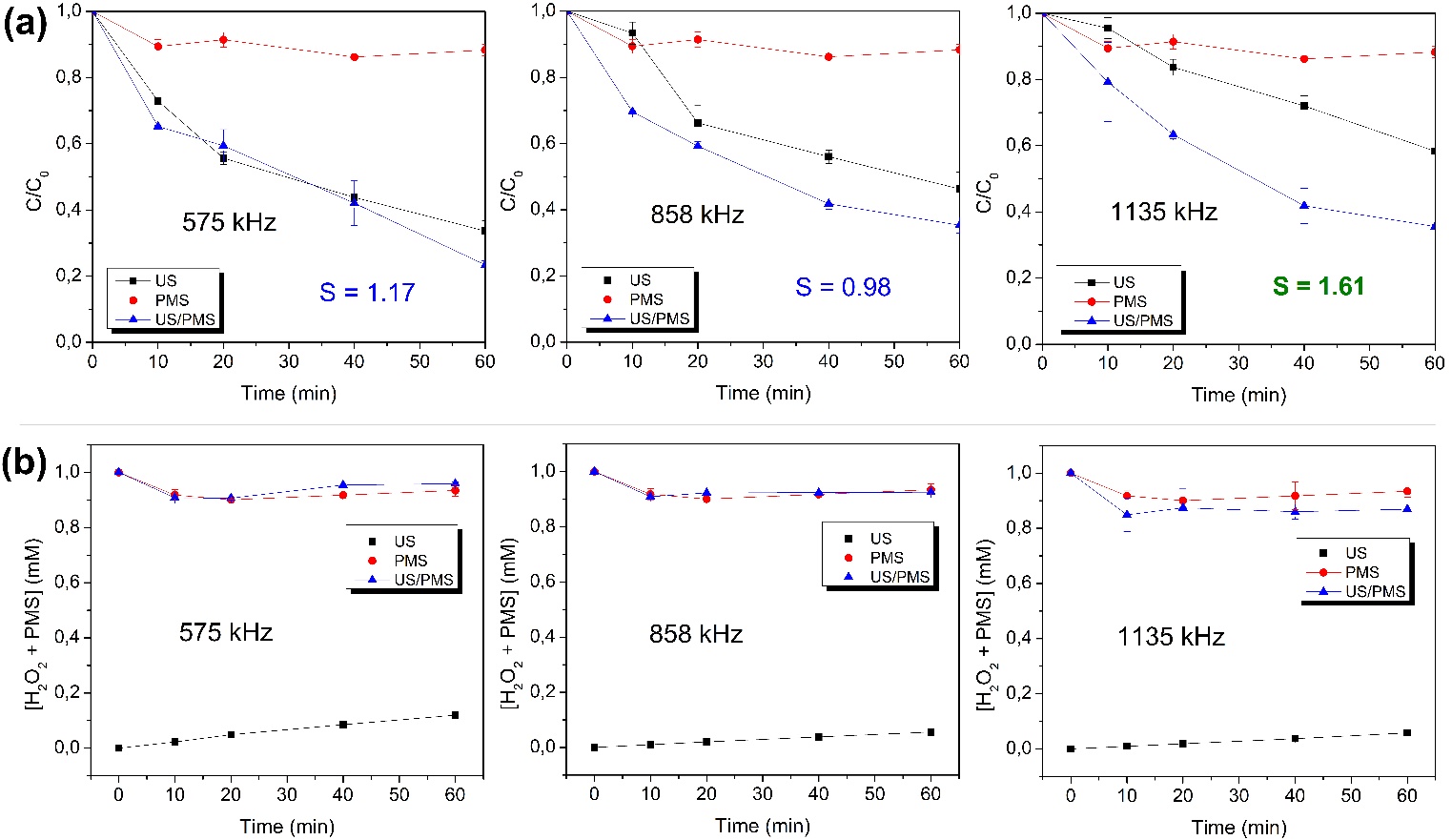


Fig. S3. Effect of the ultrasonic frequency. Degradation of ACE by US (black curves), direct oxidation (red curves), and the US/PMS system (blue curves) at different frequencies: (a) C/C_0_ *vs.* degradation time, (b) Oxidant accumulation (H_2_O_2_ + PMS). Conditions: [ACE]: 40 µM in DW, [PMS]: 1 mM, V: 360 mL, pH_initial_: 5.86 (ACE), 3.18 (ACE + PMS), frequencies of 575, 858, and 1135 kHz, with power densities of 84.14, 87.07, and 85.81 W L^–1^, respectively.


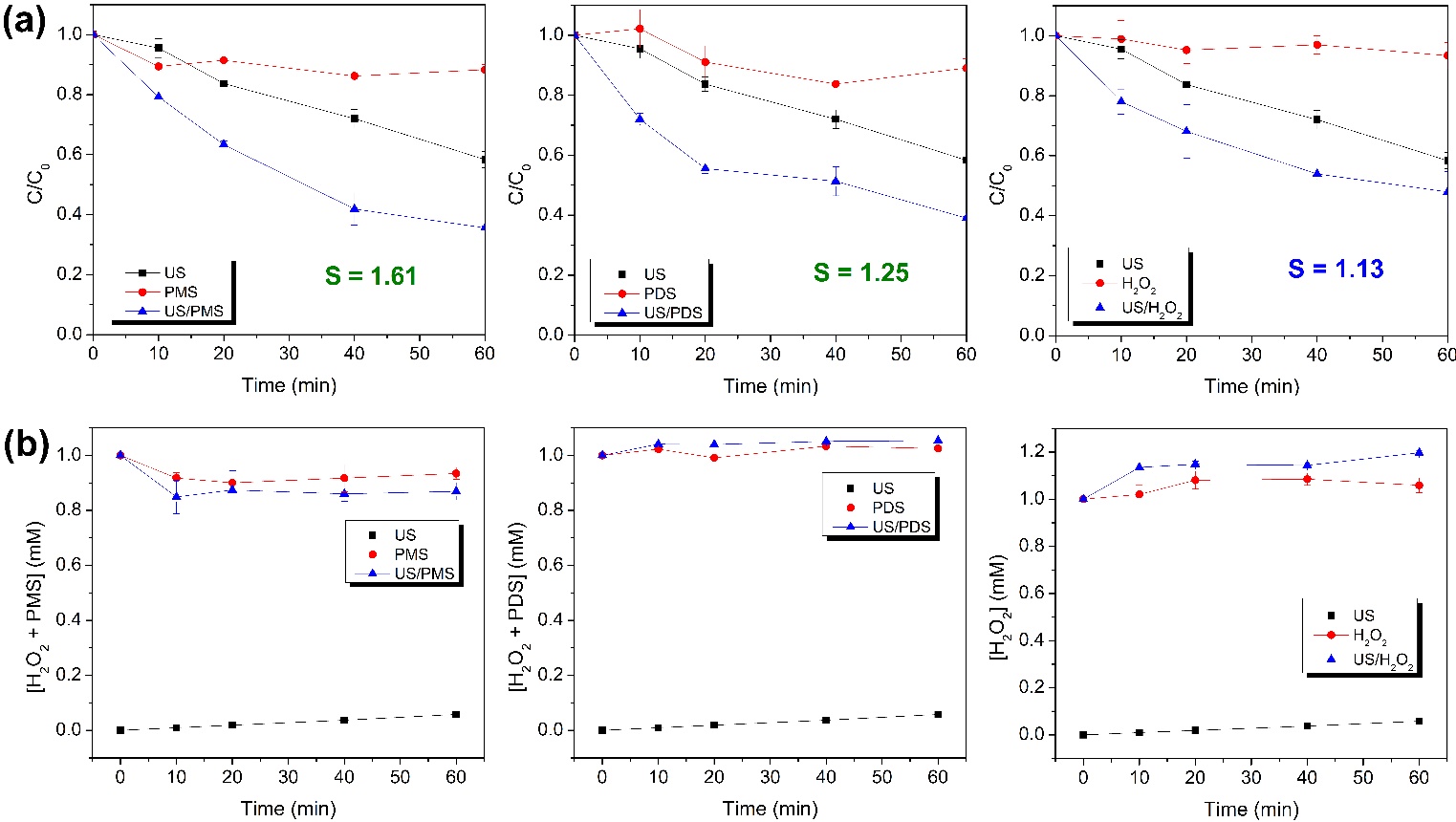


Fig. S4. Effect of the type of oxidant. Degradation of ACE by US (black curves), direct oxidation (red curves), and the US/Oxidant system (blue curves) at 1135 kHz: (a) C/C_0_ *vs.* degradation time, (b) Oxidant accumulation (H_2_O_2_ + PMS or PDS). Conditions: [ACE]: 40 µM in DW, [Oxidant]: 1 mM, V: 360 mL, pH_initial_: 5.86 (ACE), 3.18 (ACE + PMS), 5.72 (ACE + PDS), 5.77 (ACE + H_2_O_2_), frequency: 1135 kHz, power density: 85.81 W L^–1^.


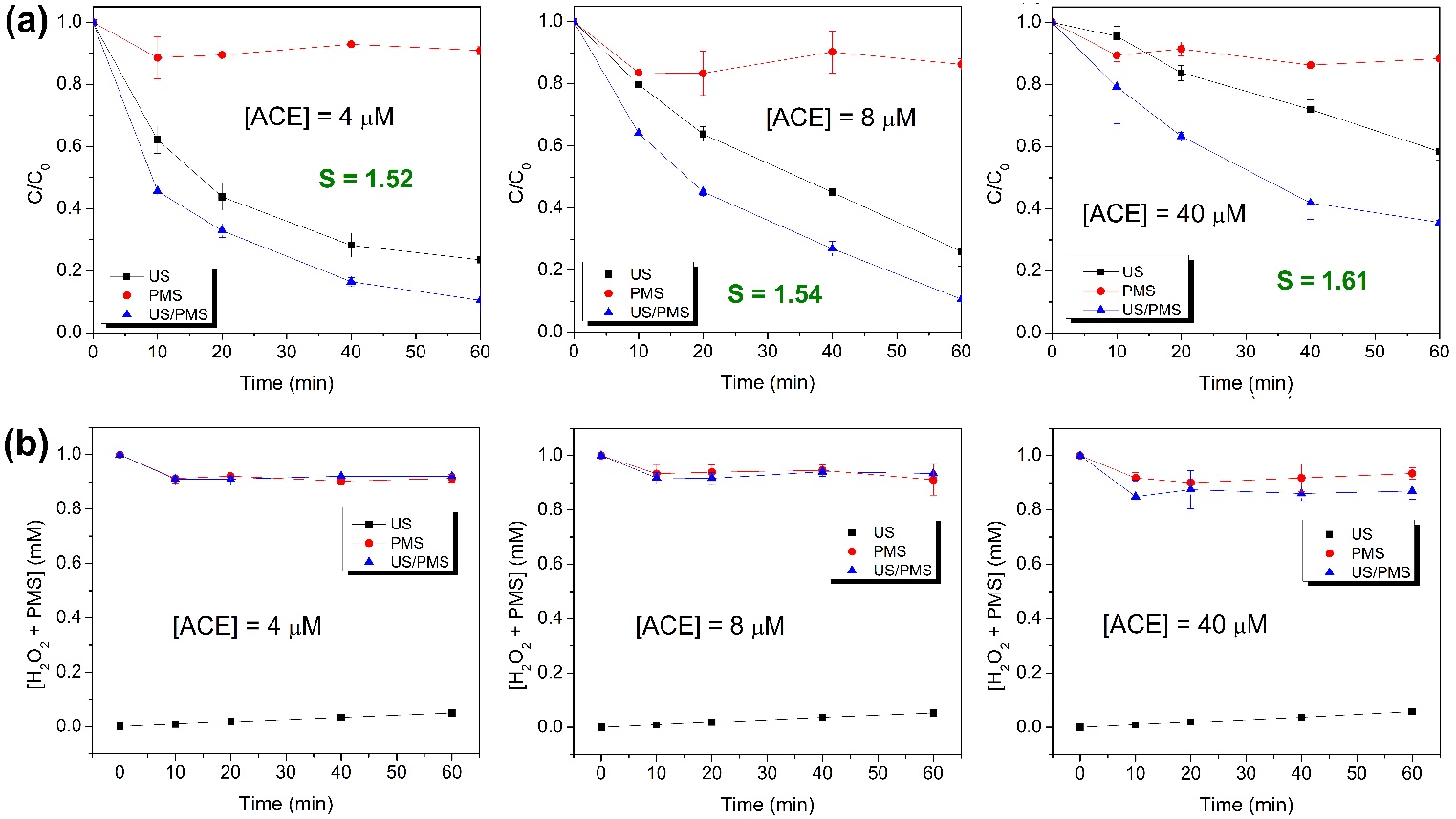


Fig. S5. Effect of initial ACE concentration. Degradation of ACE by US (black curves), direct oxidation (red curves), and the US/PMS system (blue curves) at 1135 kHz: (a) C/C_0_ *vs.* degradation time, (b) Oxidant accumulation (H_2_O_2_ + PMS). Conditions: [ACE]: 4, 8, and 40 µM in DW, [PMS]: 1 mM, V: 360 mL, pH_initial_: 5.77-5.86 (ACE 4-40 µM), 3.18 (ACE 4-40 µM + PMS), frequency: 1135 kHz, power density: 85.81 W L^–1^.


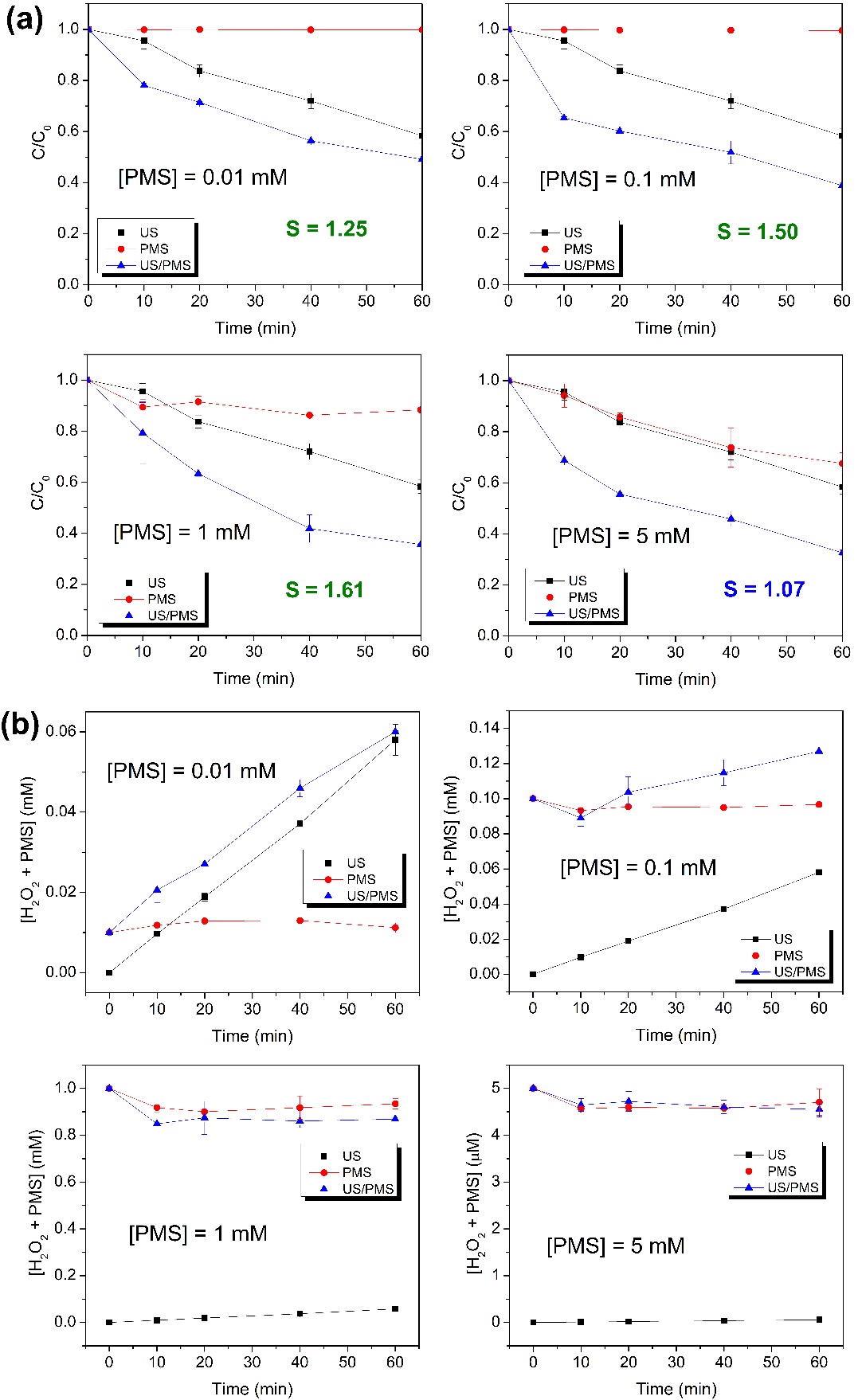


Fig. S6. Effect of oxidant concentration. Degradation of ACE by US (black curves), direct oxidation (red curves), and the US/PMS system (blue curves) at 1135 kHz: (a) C/C_0_ *vs.* degradation time, (b) Oxidant accumulation (H_2_O_2_ + PMS). Conditions: [ACE]: 40 µM in DW, [PMS]: 0.01, 0.1, 1, and 5 mM, V: 360 mL, pH_initial_: 5.86 (ACE), 5.25 (ACE + PMS 0.01 mM), 4.16 (ACE + PMS 0.1 mM) 3.18 (ACE + PMS 1 mM), 2.69 (ACE + PMS 5 mM), frequency: 1135 kHz, power density: 85.81 W L^–1^.

**Supplementary references**

Botero-Coy AM, Martínez-Pachón D, Boix C, et al (2018) ‘An investigation into the occurrence and removal of pharmaceuticals in Colombian wastewater.’ Sci Total Environ 642:842–853. https://doi.org/10.1016/j.scitotenv.2018.06.088

Ferkous H, Merouani S, Hamdaoui O, Pétrier C (2017) Persulfate-enhanced sonochemical degradation of naphthol blue black in water: Evidence of sulfate radical formation. Ultrason Sonochem 34:580–587. https://doi.org/10.1016/j.ultsonch.2016.06.027

Fisher ES, Curry SC (2019) Evaluation and treatment of acetaminophen toxicity. In: Ramachandran A, Jaeschke HBT-A in P (eds) Drug-induced Liver Injury. Academic Press, pp 263–272

Kimura T, Sakamoto T, Leveque J-M, et al (1996) Standardization of ultrasonic power for sonochemical reaction. Ultrason Sonochem 3:S157–S161. https://doi.org/10.1016/S1350-4177(96)00021-1

Liang C, He B (2018) A titration method for determining individual oxidant concentration in the dual sodium persulfate and hydrogen peroxide oxidation system. Chemosphere 198:297–302. https://doi.org/10.1016/j.chemosphere.2018.01.115

Liang C, Huang C-F, Mohanty N, Kurakalva RM (2008) A rapid spectrophotometric determination of persulfate anion in ISCO. Chemosphere 73:1540–1543. https://doi.org/10.1016/j.chemosphere.2008.08.043

Patel M, Kumar R, Kishor K, et al (2019) Pharmaceuticals of Emerging Concern in Aquatic Systems: Chemistry, Occurrence, Effects, and Removal Methods. Chem Rev 119:3510–3673. https://doi.org/10.1021/acs.chemrev.8b00299

Phong Vo HN, Le GK, Hong Nguyen TM, et al (2019) Acetaminophen micropollutant: Historical and current occurrences, toxicity, removal strategies and transformation pathways in different environments. Chemosphere 236:124391. https://doi.org/10.1016/j.chemosphere.2019.124391

Serna-Galvis EA, Silva-Agredo J, Giraldo-Aguirre AL, Torres-Palma RA (2015) Sonochemical degradation of the pharmaceutical fluoxetine: Effect of parameters, organic and inorganic additives and combination with a biological system. Sci Total Environ 524–525:354–360. https://doi.org/10.1016/j.scitotenv.2015.04.053

Vulliet E, Cren-Olivé C (2011) Screening of pharmaceuticals and hormones at the regional scale, in surface and groundwaters intended to human consumption. Environ Pollut 159:2929–2934. https://doi.org/10.1016/j.envpol.2011.04.033

Wang S, Zhou N (2016) Removal of carbamazepine from aqueous solution using sono-activated persulfate process. Ultrason Sonochem 29:156–162. https://doi.org/10.1016/j.ultsonch.2015.09.008

Zhao Y, Ye L, Zhang X-X (2018) Emerging Pollutants–Part I: Occurrence, Fate and Transport. Water Environ Res 90:1301–1322. https://doi.org/10.2175/106143018X15289915807236
